# An Iterative Deflectometry Method of Reconstruction of Separate Specular Surfaces

**DOI:** 10.3390/s25051549

**Published:** 2025-03-02

**Authors:** Cheng Liu, Jianhua Liu, Yanming Xing, Xiaohui Ao, Hongda Shen, Chunguang Yang

**Affiliations:** 1 School of Mechanical Engineering, Beijing Institute of Technology, Beijing 100081, China; 7520240040@bit.edu.cn (C.L.); Jeffliu@bit.edu.cn (J.L.); hdshen07@163.com (H.S.); 2 National Key Laboratory of Special Vehicle Design and Manufacturing Integration Technology, Beijing Institute of Technology, Beijing 100081, China; su_phoenix@163.com (Y.X.); chunguang33@126.com (C.Y.); 3 Hebei Key Laboratory of Intelligent Assembly and Detection Technology, Tangshan Research Institute, Beijing Institute of Technology, Tangshan 063000, China

**Keywords:** separate specular surfaces, deflectometry, stereoscopic PMD, B-spline surface

## Abstract

Phase measuring deflectometry (PMD) plays a more and more significant role in the measurement of specular surfaces. However, most of the deflectometric methods are only suitable for continuous specular surfaces, but not for the discontinuous surfaces. In this work, with the hardware of stereoscopic PMD, a mechanism is introduced so that a specular surface can be reconstructed iteratively with the pre-known coordinate of a reflecting point. Based on the mechanism and the excellent local properties of the B-spline surface, a reconstruction method suitable for both kinds of specular surfaces is proposed. Meanwhile, to resist the noise of the single point, this work mathematically analyzes the mechanism of the method. With the mathematical conclusion, the sparse point cloud solved using stereoscopic PMD is employed to scale the B-spline surfaces, improving the accuracy of reconstruction. Simulated and actual experiments are carried out, and the results show high accuracy and robustness of the PMD system and the reconstruction method.

## 1. Introduction

Components with specular surfaces are widely used in aerospace, optics systems, etc. There is a large number of components consisting of separate specular surfaces, not just a continuous specular surface to efficiently adjust optical paths and reduce the volume of optical instruments, such as monolithic multi-surface workpieces (MMSWs) in imaging spectrometers and off-axis telescopes [1,2,3,4]. As an effective contact measurement method, a coordinate measurement machine (CMM) can measure specular surfaces accurately but with injury to the specular surfaces [5]. As an effective non-contact measurement method, interferometry can only measure sample and small-scale specular surfaces [6]. Effective measurement technologies for components with separate specular surfaces are urgently needed. Reaching high efficiency and accuracy, phase measuring deflectometry (PMD) is a no-contact, full-field measurement technology for specular surfaces. A system of PMD usually consists of cameras and monitors. Reflected and distorted by the specular surfaces under test (SUTs), the patterns displayed by the monitors are recorded with the cameras. In a calibrated PMD system where the position and posture among the cameras and monitors are solved, from the captured distorted patterns, the three-dimensional (3-D) reconstruction of the SUT can be conducted [7]. PMD completes 3-D reconstruction with high accuracy and simple equipment, which is very competitive in the field of measuring specular surfaces [8].

At present, PMD mainly focuses on the 3-D reconstruction of the continuous SUT, achieving great success. With reprojection error on the monitor as a cost function, Refs. [9,10] models the SUT to convert the reconstruction to the optimization of the parameters of the model. Refs. [11,12] utilize binocular cameras to assess the slope distribution of the SUT. With the slope distribution, the reconstruction of the SUT is achieved through the integration of the slopes. In a PMD system of one camera, Ref. [13] reconstructs a continuous SUT through the differential geometry analysis of the surface. Recently, Ref. [14] measured the slope distribution of a continuous SUT with a telecentric imaging lens and Fourier lens, which makes the distortion of reflected patterns only sensitive to the slopes. Solving the problem of the height–slope ambiguity [15], the development of PMD in measuring a continuous SUT makes it a more and more mature technology.

When it comes to the reconstruction of separate or discontinuous specular surfaces, previous PMD schemes need a translating monitor [16,17] or more than one monitor [18,19,20]. However, the extra or translating monitors are prone to enlarge the error in the reconstruction and make the measurement difficult. Thus, PMD with fixed hardware is urgently demanded for the accurate and simple measurement of separate specular surfaces. With the fixed hardware of stereoscopic PMD (stereo-PMD), Ref. [21] models discontinuous specular surfaces with a B-spline surface; according to the slope distribution solved with stereo-PMD, it solves control points of the B-spline surface to reconstruct the SUT, improving the accuracy of reconstruction and solving the 3-D coordinate of the SUT in the camera coordinate system rather than merely the relative height of the reflecting points [22,23].

With the hardware of stereo-PMD and the coordinate of a reflecting point solved with stereo-PMD in advance, Ref. [24] introduces a mechanism of the iterative reconstruction of a continuous SUT. Because it only solves the coordinate of a single reflecting point, the method based on the mechanism is more efficient than stereo-PMD, which solves the slope distribution of all the reflecting points point by point and is followed by the integration of the slopes. However, because of the noise of the electronic devices, it is difficult to guarantee the accuracy of the single reflecting point in real measurements. The application of the mechanism in PMD should be further researched to resist the noise of the single point and extend to the separate SUT.

In this paper, a deflectometric iterative reconstruction method is proposed to increase the noise robustness of the mechanism and to extend the mechanism to the measurement of the separate SUT. Some locally defined functions, like the B-spline function, are more suitable for fitting discontinuous surfaces than the globally defined functions, such as the polynomial function. By discarding the basic functions of the B-spline surface that link the discontinuous surfaces and the corresponding control points, it is possible to represent the discontinuous surfaces using a single mathematical formulation in the form of the B-spline surface. With the SUT modeled with the B-spline surface, the mechanism is analyzed mathematically, verifying that the single reflecting point can be replaced with the sparse point cloud of the SUT to increase the robustness of the methods. With the modeled SUT, the B-spline surface makes it possible to iteratively reconstruct the separate SUT based on the mechanism. In the iteration process, the *x*, *y* components of the coordinate of reflecting points in the camera coordinate system are variant, different from the fixed *x*, *y* components calculated with stereo-PMD in Ref. [21]. Thus, according to the updated slopes, a new method of reconstructing the SUT in the form of the B-spline surface in every iteration process is proposed.

## 2. Proposed Methodology

In this section, firstly, the mechanism of the proposed method is introduced in detail. Secondly, stereo-PMD is employed to solve the 3-D coordinate of a reflecting point. Finally, combined with the model of the perspective projection camera, a B-spline surface is introduced to model the SUT. Meanwhile, the mathematical analysis of the mechanism is conducted to replace the single reflecting point with the sparse point cloud of the SUT.

### 2.1. Mechanism of the Proposed Method

**Figure 1:**
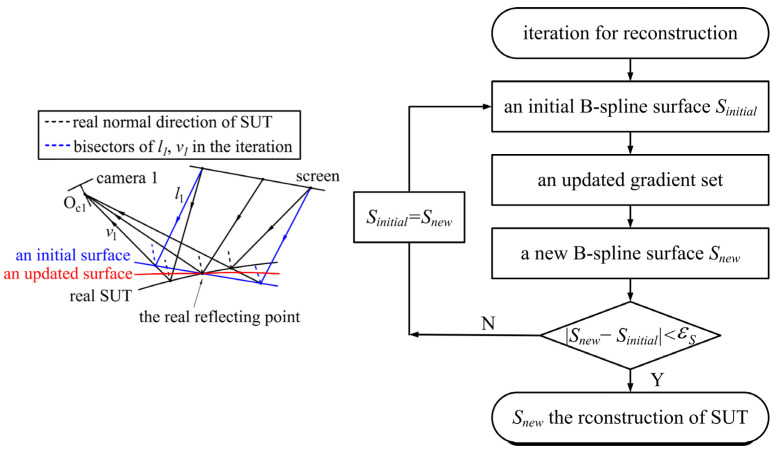
Diagram of the mechanism and procedures of the iterative reconstruction method.

As an initial surface passes through a real reflection point, the updated gradient set, calculated with the bisector of *l*_1_ and *v*_1_, is different from the gradient set of the initial surface and more approximated to the real gradient set of the SUT, as illustrated in Figure 1. Passing through the real reflection point, the updated surface reconstructed according to the updated gradient set is also closer to the SUT. With the updated surface as a new initial surface, the newly updated surface will be much closer to the SUT than the previously updated surface. Thus, as the iteration progresses, the iteratively updated surface will be an approximation of the SUT. In the reconstruction of every iteration process, a B-spline surface is employed to fit the surface according to the updated gradient set. The procedures of the iteration are illustrated in Figure 1 and listed as follows: firstly, an initial B-spline surface *S_initial_* through the point is fitted to calculate an updated gradient set; secondly, according to the updated gradient set, a new B-spline surface *S_new_* through the points is updated; finally, the B-spline surface is updated iteratively according to the first and second steps, until the variation of the 3-D coordinate |*S_new_* − *S_initial_|* between the two surfaces in one iteration process is less than a preset variation threshold $\varepsilon_{S}$. When the iteration process terminates, the B-spline surface *S_new_* is the reconstruction of the SUT. The 3-D coordinate of the real reflecting point is solved with stereo-PMD; the reconstruction at each iteration process is based on the perspective projection camera model and the differentiation properties of the B-spline surface.

### 2.2. Stereo-PMD for a Reflecting Point

The proposed method employs stereo-PMD to calculate the coordinate of a reflection point on every separate surface of the SUT, which the updated surfaces pass through. In the measurement of the SUT, two cameras record reflected fringe patterns of the SUT, which are displayed with the monitor and selected with the technique of fringe pattern analysis [25,26].

**Figure 2:**
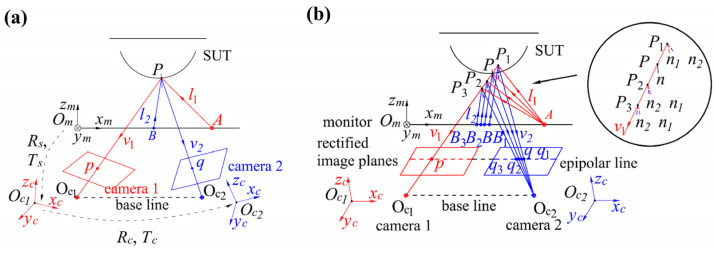
Diagram of matching homologous pixels in a rectified image pair. (**a**) Calibrated stereo-PMD system; (**b**) rectified stereo-PMD system.

After the calibration of the stereo-PMD system, the intrinsic matrixes *K_i_* (*i* = 1, 2) of the cameras, the rotation matrix *R_c_*, and translating vector *T_c_* between the camera coordinate systems (*O_c_*-*x_c_*-*y_c_*-*z_c_*), the *R_m_* and *T_m_* between camera 1 and the monitor coordinate system (*O_m_*-*x_m_*-*y_m_*-*z_m_*) are obtained, as illustrated in Figure 2a. According to *R_c_* and *T_c_*, images of the cameras are rectified [27] to make the epipolar lines and rows of pixels colinear, as illustrated in Figure 2b where the procedure of solving a reflecting point is conducted. According to *R_m_*, *T_m_*, and the rotation matrix of the rectification, the pixel coordinate on the monitor is transformed into the coordinate system of the rectified camera 1 in Figure 2b. With the unwrapped phase [25,26], the correspondence between the pixels of the cameras and the monitor can be established, such as *p* and *A*, *q* and *B*. Based on the law of reflection, the bisectors *n_i_* (*i* = 1, 2) of the incident and emergent lights *l_i_*, *v_i_* should be coincident at the reflection point *P*, which is the normal direction *n* of the SUT at *P*. It is the criterion to match the base integer pixel *p* with its homologous pixel *q*. Along the epipolar line (the same row of *p* in camera 2), sub-pixel coordinate *q*, which corresponds to the largest consistency of the bisectors among *q* and *q_j_* (*j* = 1, 2, 3), is the sub-pixel homologous pixel of *p*. The iterative matching method in [11] can be applied to match *q* and *p*. With *q* and *p*, the coordinate of *P*(*x_c_*, *y_c_*, *z_c_*) can be calculated with triangulation.

### 2.3. Perspective Projection Model

**Figure 3:**
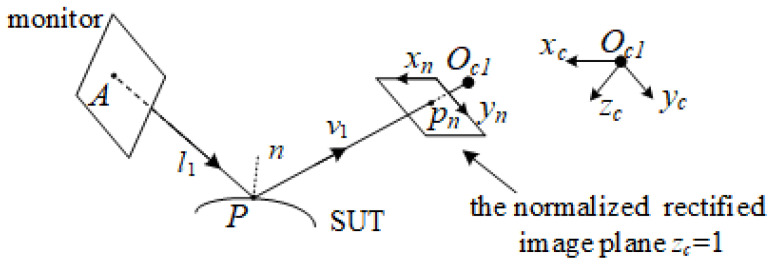
Perspective projection camera model in deflectometry.

In the perspective camera model in Figure 3, for every pixel *p* in camera 1, *p_n_* = *K*_1_^–1^ [*p* 1]^T^ = [*x_n_ y_n_* 1]^T^ is the coordinate of *p* on the normalized image plane. The coordinate of the reflection point *P* can also be expressed as *s*(*x_n_*, *y_n_*)*p_n_*, where *s*(*x_n_*, *y_n_*) is equal to the *z* component of *P* (the depth of *P*). Thus, the reconstruction of the updated surfaces is equivalent to solving the value *s* of *p*. The relationship between the gradient set $\left(\partial z_{c}/\partial x_{c},\partial z_{c}/\partial y_{c}\right)$ and the slope set $\left(\partial s/\partial x_{n},\partial s/\partial y_{n}\right)$, which, respectively, describe the slopes of the point *P* in the camera coordinate system and the slope of *s* on the normalized image plane, can be expressed as follows [21]:

(1) $$\left\{\begin{array}{l} \frac{\partial s}{\partial x_{n}}=\frac{s\frac{\partial z_{c}}{\partial x_{c}}}{1-x_{n}\frac{\partial z_{c}}{\partial x_{c}}-y_{n}\frac{\partial z_{c}}{\partial y_{c}}}\\ \frac{\partial s}{\partial y_{n}}=\frac{s\frac{\partial z_{c}}{\partial y_{c}}}{1-x_{n}\frac{\partial z_{c}}{\partial x_{c}}-y_{n}\frac{\partial z_{c}}{\partial y_{c}}} \end{array}\right..$$

### 2.4. B-Spline Surface

In the proposed iterative method, *s*(*x_n_*, *y_n_*) are modeled with a B-spline surface in the form of *S* = *WC*, where *S* is a column vector with every element corresponding to the depth *s* of every reflection point, *C* the column vector of the control points of the B-spline surface, and *W* a parameters matrix with the elements of every row corresponding to the values of the B-spline basis functions at (*x_n_*, *y_n_*). For the *k*th element *S*(*k*) of *S*, the coordinate of *P* in the camera coordinate system is *S*(*k*) *p_n_*(*x_n_*, *y_n_*, 1). The task of reconstruction at each iteration process is to solve *C* according to the updated gradient set.

Partial derivatives of *s*(*x_n_*, *y_n_*), denoted as $\partial S/\partial x_{n}$ and $\partial S/\partial y_{n}$, in the form of B-spline surfaces are B-spline surfaces with the basic functions one order lower than *s*(*x_n_*, *y_n_*) in the directions of *x_n_* and *y_n_*, respectively. The column vectors of the control points of $\partial S/\partial x_{n}$ and $\partial S/\partial y_{n}$ are denoted as *C_x_* and *C_y_*, respectively. For the B-spline surfaces, the parameters matrices *D_x_*, *D_y_* describe the inherent relationship between *C* and *C_x_*, *C_y_*, respectively. Thus, $\partial S/\partial x_{n}$, $\partial S/\partial y_{n}$ can be expressed as

(2) $$\left\{\begin{array}{l} \frac{\partial S}{\partial x_{n}}=W_{x}C_{x}=W_{x}D_{x}C\\ \frac{\partial S}{\partial y_{n}}=W_{y}C_{y}=W_{y}D_{y}C \end{array}\right.,$$

where *W_x_*, *W_y_*, respectively, denote the parameters matrices of the surfaces. Substituting Equation (2) into Equation (1),

(3) $$\left(\begin{array}{l} W_{x}D_{x}-\frac{\partial z_{c}}{\partial x_{c}}.*\frac{1}{1-X_{n}.*\frac{\partial z_{c}}{\partial x_{c}}-Y_{n}.*\frac{\partial z_{c}}{\partial y_{c}}}.*W\\ W_{y}D_{y}-\frac{\partial z_{c}}{\partial y_{c}}.*\frac{1}{1-X_{n}.*\frac{\partial z_{c}}{\partial x_{c}}-Y_{n}.*\frac{\partial z_{c}}{\partial y_{c}}}.*W \end{array}\right)C=0$$

is derived in the form of *AC* = 0, where *X_n_*, and *Y_n_*, respectively, denote column vectors of *x_n_* and *y_n_*; ‘.*’ denotes the Hadamard product. Because the rank of *A* minus its number of columns is −1 [21], the explicit solution of *C* should be scaled to *C*·*s_k_real_*/*S*(*k*), where *s_k_real_* is a pre-known depth of *S*(*k*) calculated with previous stereo-PMD.

In Section 2.1, the mechanism is introduced with the one real reflecting point. The conclusion of the rank of *A* makes it rational to scale *C* with more than one reflecting point, such as the sparse point cloud of the SUT, resulting in higher noise robustness. The sparse point cloud scales *C* to

(4) $$C=C\cdot \sum_{k=1}^{K}\left[s_{k}/KS(k)\right],$$

where *K* is the number of points in the cloud, and *s_k_* is the calculated depth of the *k*th point in the cloud with stereo-PMD.

If the SUT with *n* separate specular surfaces is measured, *S* = *WC* will be in the form of (*S*_1_; …; *S_n_*)′ = (*W*_1_; …; *W_n_*)′(*C*_1_; …; *C_n_*)′, which distinguishes separate surfaces automatically for the local definition of the B-spline surfaces. The method of constructing *W* of discontinuous surface refers to Ref. [21]. *n* points with known depths, respectively, on the surfaces or the sparse point cloud of the *n* separate surfaces can be used to scale *C*_1_, …, and *C_n_*, respectively.

## 3. Experiments

### 3.1. Simulation

**Figure 4:**
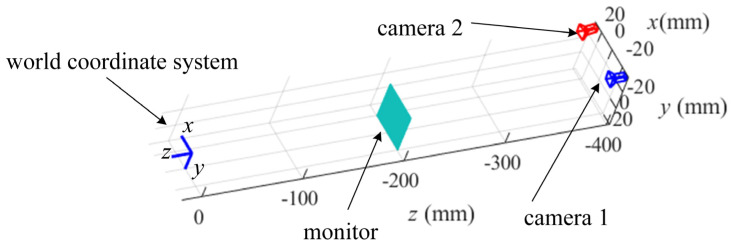
Simulated system of stereo-PMD.

**Figure 5:**
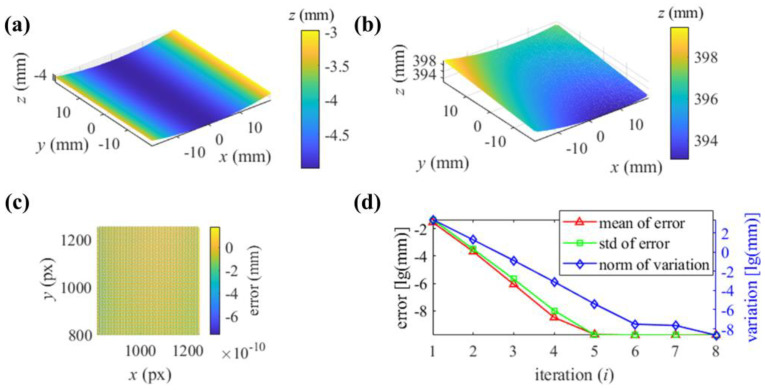
The reconstruction of the cylindrical SUT with the iterative surface passing through a real reflecting point: (**a**) the cylindrical SUT in the world coordinate system; (**b**) reconstruction by the proposed method in the camera 1 coordinate system; (**c**) depth error distribution; (**d**) process of the iteration.

**Figure 6:**
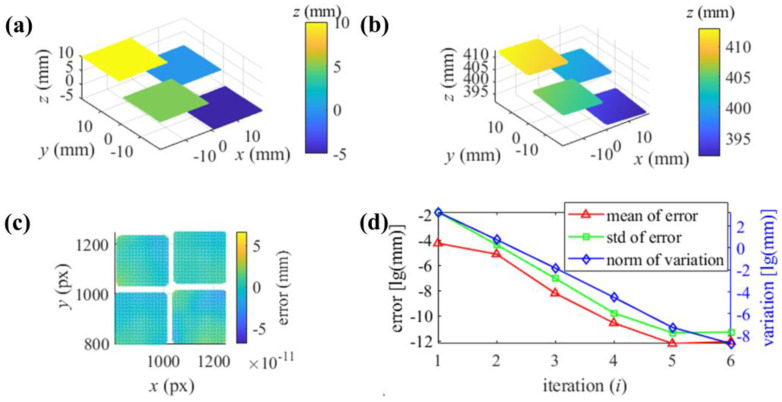
The reconstruction of the separate SUT with the iterative surface passing through real reflecting points: (**a**) the separate SUT in the world coordinate system; (**b**) reconstruction using the proposed method in the camera 1 coordinate system; (**c**) depth error distribution; (**d**) process of the iteration.

For testing the proposed reconstruction method, a simulated system of stereo-PMD is set up to conduct experiments with the camera resolution 2048 × 2048 and pixel size 3.45 µm × 3.45 µm, as illustrated in Figure 4. In the simulation, the SUT of a cylinder with a radius of 100 mm and the SUT with four separate parallel specular planes with a distance of 5 mm are measured separately. Figure 5a and Figure 6a illustrate the SUTs in the world coordinate system, respectively. The corresponding relationship between the pixels of the cameras and the monitor is established by the spatial geometric relationship between the cameras and the monitor. With an adjacent knot interval of 5 pixels, the B-spline surfaces of cubic basis functions are used to model the SUT. The stereo-PMD solves the coordinate of a real reflecting point and the sparse point cloud of the SUT. The proposed iterative reconstruction method is conducted in the coordinate system of camera 1.

In testing the proposed iterative method, with the initialization as a plane, the updated B-spline surface passes through the reflecting points with their real coordinates. The reconstruction of the cylinder in the coordinate system of camera 1 is illustrated in Figure 5b. With every pixel of camera 1 corresponding to the depth of a reflecting point, the error of depth is illustrated in Figure 5c. From Figure 5c, the root mean square error (rmse) of the iterative reconstruction is 1.6904 × 10^−10^ mm. The mean value and standard deviation (std) of the depth error in every iteration process and the norm of the depth variation of every updated surface are illustrated in Figure 5d.

In measuring the separate SUT, four reflecting points with the real coordinates, respectively, on the planes are used to scale *C*_1_, …, and *C*_4_, respectively. The reconstruction of the SUT, the error of the depth, and the process of iteration are illustrated in Figure 6b–d, respectively. From Figure 6c, the rmse of the iterative reconstruction is 4.8694 × 10^−12^ mm. The accuracy of the iterative reconstruction method and its mechanism can be verified with the simulation.

**Figure 7:**
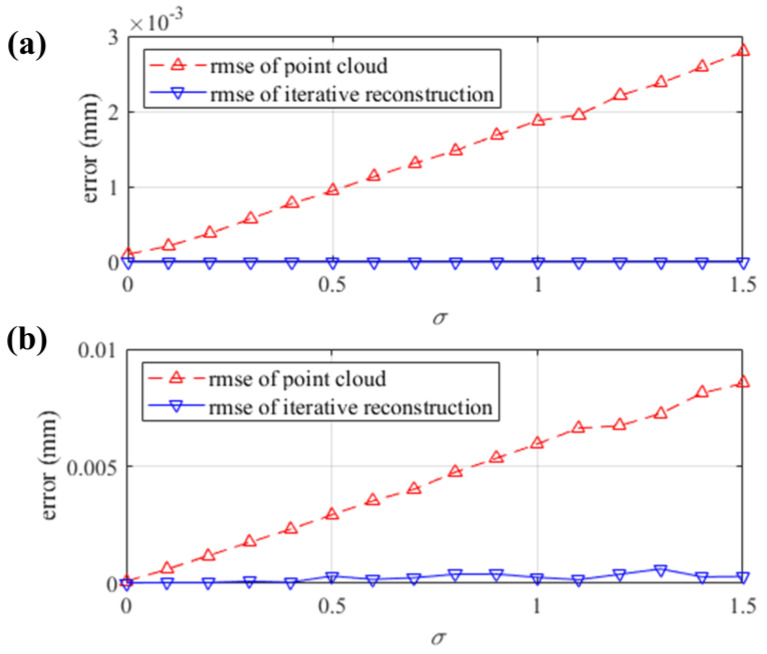
The depth error of the sparse point cloud and the iterative reconstruction. (**a**) Error in the reconstruction of the cylindrical SUT; (**b**) error in the reconstruction of the separate SUT.

However, disturbed by noise, the real coordinates of the reflection points can be obtained in actual measurement. With the std of image intensity around 126, the ideal fringe patterns are generated by the spatial geometric relationship of the simulated system. With the std σ varying from 0 to 1.5, two-dimensional Gaussian noise is introduced to the ideal fringe patterns. The minimum signal-to-noise ratio of the noisy patterns is around 19.2 dB. With the noisy patterns, the simulated stereo-PMD is employed to obtain the sparse point cloud of the SUTs with the base pixel interval of 10 pixels (pxs). The control points of the B-spline surfaces in every iteration process are scaled by the point cloud according to Equation (4). Figure 7 shows the rmse of the sparse point cloud and the iterative reconstruction compared to the ground truth of the simulation, demonstrating the robustness of the iterative method.

**Figure 8:**
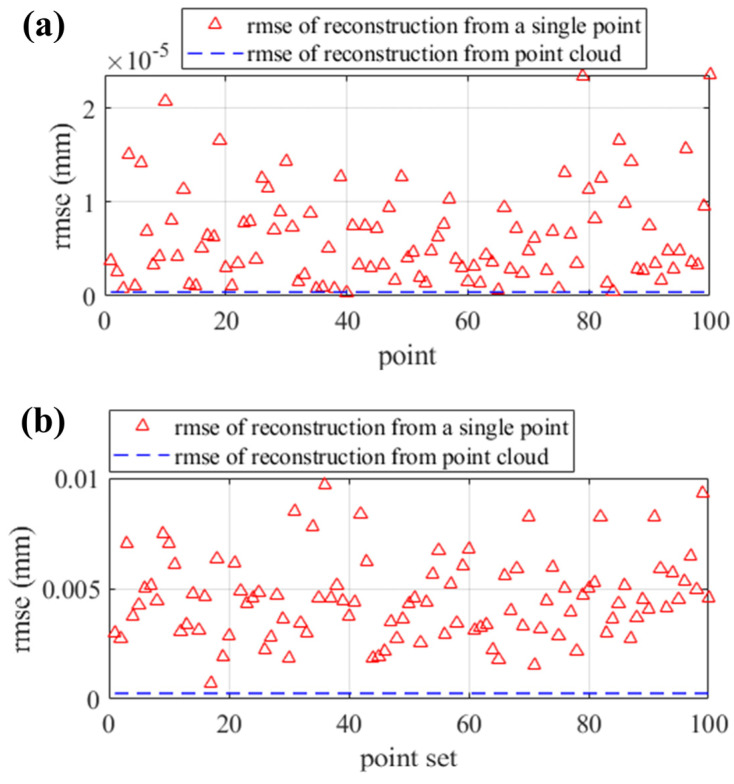
The comparisons between reconstruction from a single point and the point cloud. (**a**) Error in the reconstruction of the cylindrical SUT; (**b**) error in the reconstruction of the separate SUT.

To verify that the iterative method utilizing the sparse point cloud provides more reliable reconstruction than a single point, a total of one hundred comparisons are conducted between reconstruction from a single point and the point cloud. For the cylindrical SUT, 100 randomly selected points are utilized to scale the control points, respectively, in 100 different iterative reconstructions. For the separate SUT, 100 sets of points are randomly selected to scale the control points, with each set consisting of four points located on the four surfaces. Under Gaussian noise with a std of 1, the depth errors of the iterative reconstruction with the sparse point cloud and the single point are illustrated in Figure 8. Figure 8 demonstrates that the iterative reconstruction from the point cloud is more reliable than using a single point.

### 3.2. Actual Measurement

To verify the proposed method in actual measurement, a system of stereo-PMD was set up with two cameras (DAHENG IMAGING, MER2-503-36U3M, resolution: 2448 × 2048, pixel size: 3.45 µm × 3.45 µm) and a computer monitor (DELL E1715S). With the assistance of a plane mirror, the system was calibrated with the calibration methods in [11,17] to obtain *K_i_* (*i* = 1, 2), *R_c_*, *T_c_*, *R_m_*, and *T_m_*.

**Figure 9:**
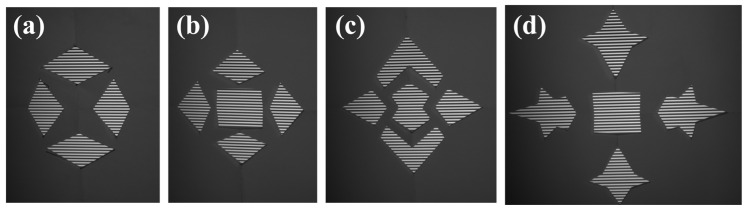
Horizontal fringe patterns, respectively, reflected with four SUTs and recorded with camera 1. (**a**) Reflected pattern with SUT 1; (**b**) reflected pattern with SUT 2; (**c**) reflected pattern with SUT 3; (**d**) reflected pattern with SUT 4.

**Figure 10:**
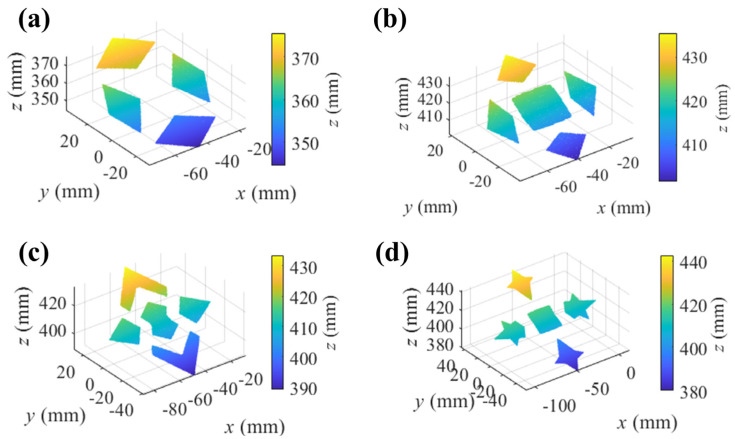
Reconstruction of SUTs. (**a**) Reconstruction of SUT 1; (**b**) reconstruction of SUT 2; (**c**) reconstruction of SUT 3; (**d**) reconstruction of SUT 4.

In the measurement, the monitor displayed fringe patterns in horizontal and vertical directions, separately. The reflected patterns of the SUTs were recorded with the cameras. In the measurement, four SUTs were measured, separately. Figure 9 illustrates the reflected fringe patterns of the horizontal direction recorded with camera 1. With the unwrapped phase maps, the stereo-PMD solved the sparse point cloud of the SUTs. The plane mirror at one of the poses in the calibration was treated as an initial surface. Scaled by the point cloud, the proposed method iteratively reconstructed the SUTs, as illustrated in Figure 10. Measured by an interferometer, the separate surfaces of the measured SUTs are coplanar with a residual error of less than one micrometer. The reconstruction of the SUTs was, respectively, fitted with planes with rmse 0.0066 mm, 0.0057 mm, 0.0093 mm, and 0.0146 mm towards the fitted planes. This level of accuracy surpasses that of the sparse point cloud, which exhibited rmse values of 0.0193 mm, 0.0407 mm, 0.0281 mm, and 0.0370 mm.

The reconstruction of every single separate surface of all the SUTs was fitted with planes, respectively. For all the SUTs, the rmse of the separate fitting remains at the level of 1 × 10^−5^–1 × 10^−4^ mm. For all the SUTs, the angles among the normal directions of the planes of the separate fitting were with a mean value of 0.0043° and std 0.0026°, which were more accurate than the sparse point cloud with a mean value of 0.0958° and std 0.0685°.

The system of PMD can reach higher precision in measuring the similar specular surface in Ref. [17] with the rmse 0.0240 mm, which measures the SUTs with a PMD system of a translating monitor. The proposed method can successfully reconstruct the separate specular surface with accuracy in the 3-D coordinate and the angles of the surfaces. Meanwhile, the accuracy of the reconstruction with the stereo-PMD can be improved using the proposed method.

## 4. Conclusions

In this paper, an iterative reconstruction method of reconstructing separate specular surfaces is proposed. The method and its mechanism are introduced in detail and verified in the simulated and the actual measurements. Different from the traditional stereoscopic deflectometry, which integrally reconstructs surfaces with the fixed x, y components of the coordinate and the slope of every reflection point, the proposed method updates surfaces with the variant x, y components according to the updated slopes. Compared with the point cloud calculated with stereoscopic deflectometry, the proposed method reconstructs surfaces more accurately, which is verified in the simulated and the actual measurements. In the future, greater emphasis will be placed on the stability of the method and the optimal parameters of the deflectometric system, such as the sampling step and the focus length of the cameras, to improve the stability and accuracy of the method.

## Data Availability

Data underlying the results presented in this paper are not publicly available at this time but may be obtained from the authors upon reasonable request.
